# A Prognostic Nomogram for Postoperative Bone Remodeling in Patients with ADDWoR

**DOI:** 10.1038/s41598-018-22471-x

**Published:** 2018-03-12

**Authors:** Xiaohan Liu, Pei Shen, Xiangyu Wang, Shanyong Zhang, Jiawei Zheng, Chi Yang

**Affiliations:** 1 College of Stomatology, Shanghai Jiao Tong University School of Medicine, Shanghai, China; 2 Department of Oral Surgery, Ninth People’s Hospital, Shanghai Jiao Tong University School of Medicine, Shanghai, China; 3 Department of Oral-Maxillofacial Head and Neck Surgery, Ninth People’s Hospital, Shanghai Jiao Tong University School of Medicine, Shanghai, China; 4 Shanghai Key Laboratory of Stomatology & Shanghai Research Institute of Stomatology; National Clinical Research Center of Stomatology, Shanghai, China

## Abstract

This study aimed to establish an effective prognostic nomogram for predicting the probability of postoperative bone remodeling of patients with anterior disc displacement without reduction (ADDWoR). The nomogram was based on a retrospective study on patients underwent surgical approaches for ADDWoR at Shanghai Ninth People’s Hospital, Shanghai Jiao Tong University from January, 2007 to January, 2017. A multivariate logistic regression analysis was used to develop variables suitable for probability estimation model. The predictive accuracy and discriminative ability were determined by ROC (AUC-index) and calibration curve. Results were validated using bootstrap resampling with all statistical tests two-sided. 1110 patients were included in the analysis. The probability of postoperative bone remodeling in ADDWoR was 0.51. Six independent prognostic factors including age of onset, nocturnal bruxism, disc morphology, BMD, Wilkes’ classification, and postoperative splint therapy were integrated to construct the nomogram. The probability estimation model showed good discrimination in both internal and external validation with AUC-index of 0.84. The calibration curves for probability of postoperative bone remodeling showed optimal agreement with actual observation. In conclusion, a nomogram was established to provide individual prediction of postoperative bone remodeling for patients with ADDWoR treated by arthroscopy surgery.

## Introduction

Temporomandibular joint (TMJ) disc displacement is a common disruption condition causing progressive joint dysfunction including arthralgia (pain), functional limitation, osteoarthritis (OA), and condylar resorption^1,2^. In general population, an estimated of 36% is reported of the internal derangement (ID), which usually occurs in people of all ages and with a prevalence in female aged twenties years old^3,4^. As early as 1966, Boering *et al*. found that disc displacement might lead to condylar resorption, thus causing maxillofacial abnormalities like mandibular deviation (MD), and mandibular retrusion (MR)^5,6^. According to Schwartz *et al*., disc abnormality interrupted the growth of mandibular ramus thus resulting in maxillofacial abnormalities^7^. In their research, they included 128 patients with ADDWoR, and discovered that 112 cases reported of various degrees of facial deviation. Correspondingly, among the 60 cases of mandibular retraction, ADDWoR was observed in 56 patients. And therefore, disc displacement without reduction, especially combined with significant clinical symptoms such as pain, dysfunction, and maxillofacial abnormalities or failed to conservative therapies is general indication for invasive intervention treatment^8,9^.

According to Wolford *et al*., the replaced disc was conducive to the prevention of further resorption and degeneration of condyle and meanwhile contribute to new bone formation, especially among young patients^10^. Meanwhile, the post-operative bone regeneration which deemed as an important index of the treatment effect can relieve or reverse maxillofacial abnormalities caused by ADDWoR. For years, correlative factors involving age, gender, genetic background, nutritional status, drugs abuse, repetitive oral habits, and treatment strategy have all been cited as triggers or aggravators for the postoperative evaluation^11^. However, none of the parameters mentioned above have been specifically developed for the postoperative prognostic prediction. At the moment, nomogram, an intuitive graph of a statistical predictive model, has been proposed as an alternative standard to guide treatment allocation for pathema by formalizing and interpreting potential factors for probability prognoses^12^. Thus, a prognostic nomogram model was conducted to assess individual prognostic risk factors in estimating the probability of postoperative bone remodeling for patients with ADDWoR based on multivariate analysis.

## Results

### Clinicopathological characteristics of patients

Of the 1110 patients that underwent arthroscopy surgery for ADDWoR between 2007 and 2017, 740 patients were in the primary set and 370 in the validation set. The detailed characteristics of selected patients are listed in Table 1.

**Table 1:** Demographics and Clinicopathologic Characteristics of Patients with ADDWoR.

Demographic or Characteristic	All Patients (n = 1110)		Validation Cohort (n = 370)		Primary Cohort (n = 740)	
	No. of Patients	%	No. of Patients	%	No. of Patients	%
Gender						
Male	370	33.33	102	27.57	268	36.22
Female	740	66.67	268	72.43	472	63.78
Age, years						
Median	20.63 ± 6.20		20.31 ± 4.62		20.78 ± 6.17	
Course of disease, months						
Median	17.39 ± 16.23		17.03 ± 16.36		17.58 ± 16.24	
Nocturnal bruxism						
Yes	610	54.95	196	52.97	414	55.95
No	500	45.05	174	47.03	326	44.05
Clicking						
Yes	944	85.05	298	80.54	646	87.30
No	166	14.95	72	19.46	94	12.70
Pain						
Yes	579	52.16	194	52.43	385	52.03
No	531	47.84	176	47.57	355	47.97
MIO, mm						
Median	32.69 ± 0.88		30.05 ± 0.93		33.02 ± 0.84	
Disc morphology						
Normal	419	37.74	119	32.16	300	40.54
Depression	522	47.03	202	54.60	320	43.24
Perforation	169	15.23	49	13.24	120	16.22
BMD						
Normal	692	62.34	238	64.32	454	61.35
Osteopenia	418	37.66	132	35.68	286	38.65
Wilkes’ classification						
III	549	49.46	179	48.39	370	50.00
IV	403	36.31	141	38.10	262	35.41
V	158	14.23	50	13.51	108	14.59
Splint therapy						
Yes	345	31.08	109	29.46	236	31.89
No	765	68.92	261	70.54	504	68.11
Bone remodeling						
Yes	533	48.02	172	46.49	361	48.78
No	577	51.98	198	53.51	379	51.22

MIO, maximum interincisal opening; BMD, bone mineral density.

The age of onset ranged from 10 to 35 years (mean, 20.63 ± 6.20 years) and the ratio of female/male was 2.00. The progress of the disease lasted for an average of 17.39 ± 16.23 months (3 to 120 month). According to physical examinations, 944 patients and 579 patients have the history of clicking and pain respectively, and nocturnal bruxism were reported in 610 patients. The MIO varied from 0 to 52 mm with a mean of 32.69 ± 0.88 mm. Based upon pre-operation evaluation, the disc perforation and osteopenia rates were 15.23% and 37.66% within the cohorts. In the Wilkes’ classification, 549 cases were in stage III, 403 in stage IV, and 158 in stage V.

### Factors Associated with Postoperative bone remodeling in Patients with ADDWoR

Multivariate logistic regression analysis was performed in this study and the outcome indicated that factors including age of onset, nocturnal bruxism, disc morphology, BMD, Wilkes’ classification, and postoperative splint therapy were associated with the bone remodeling after surgery (Table 2).

**Table 2:** Multivariate Analysis of Postoperative bone remodeling in Patients with ADDWoR.

Variable	Multivariate Analysis		
	OR	95% CI	P *value*
Age, year			
≤20	Reference		
20–25	0.39	0.25–0.59	<0.001
25–30	0.23	0.12–0.43	<0.001
≥30	0.078	0.02–0.27	<0.001
Nocturnal bruxism			
Yes	Reference		
No	7.14	4.76–10.00	<0.001
Wilkes’ classification			
III	Reference		
IV	2.09	1.10–3.96	0.024
V	0.78	0.23–2.60	0.680
Disc morphology			
Normal	Reference		
Depression	5.00	2.86–9.09	<0.001
Perforation	0.52	0.21–2.09	<0.001
BMD			
Normal	Reference		
Osteopenia	0.53	0.32–0.86	0.011
Splint therapy			
Yes	Reference		
No	1.40	0.92–2.14	0.120

MIO, maximum interincisal opening; BMD, bone mineral density; OR, Odds ratio.

**Figure 1:**
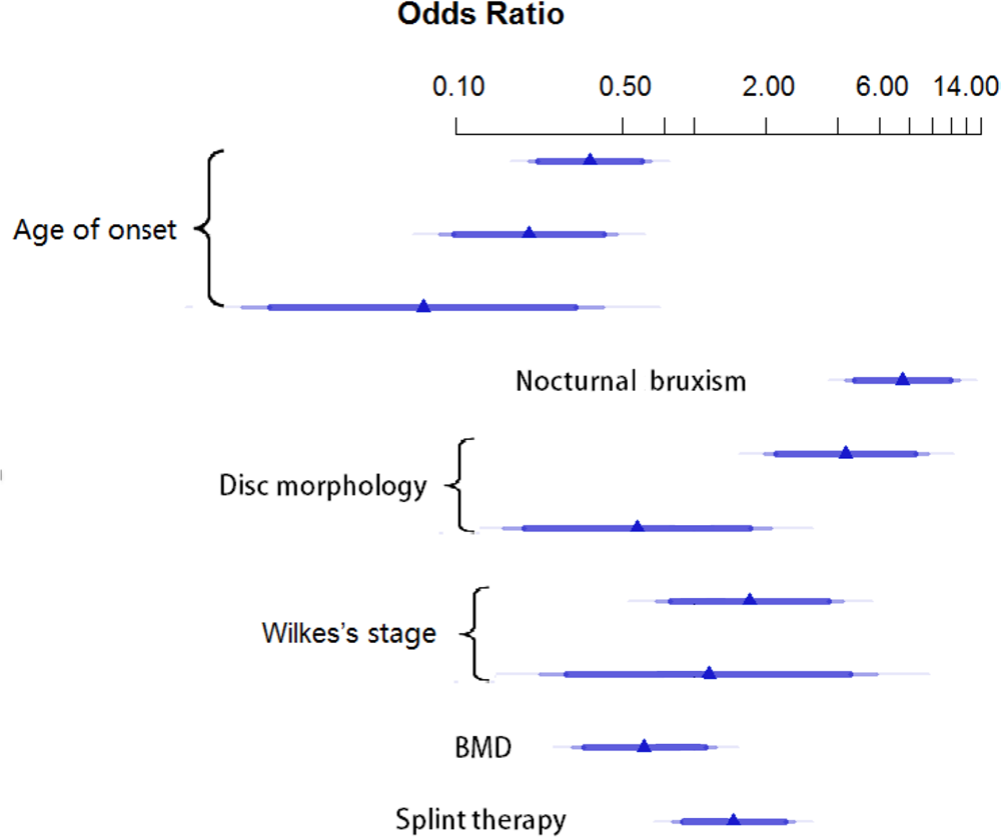
Odds ratio of multivariate analysis for postoperative bone remodeling in patients with ADDWoR.

In the analysis, onset age was the significant predictor and the post-operative bone remodeling concentrated among younger group with the OR of 0.39 (95% CI: 0.25 to 0.59, P < 0.001). According to Wilkes’ classification, patients in earlier stage achieved better outcomes (OR, 2.09; 95% CI, 1.10 to 3.96, P = 0.024). The following variables were considered as negative predictors: disc with perforation (OR, 0.08; 95% CI, 0.03 to 0.23, P < 0.001), lower BMD (OR, 0.53; 95% CI, 0.32 to 0.86, P = 0.011), and patients who have nocturnal bruxism experience (OR, 0.14; 95% CI, 0.10 to 0.21, P < 0.001). Meanwhile, a preferred result was also yield in patients with postoperative splint therapy with a OR of 1.40 (95% CI: 0.92 to 2.14, P = 0.120) (Fig. 1).

### Prognostic Nomogram

**Figure 2:**
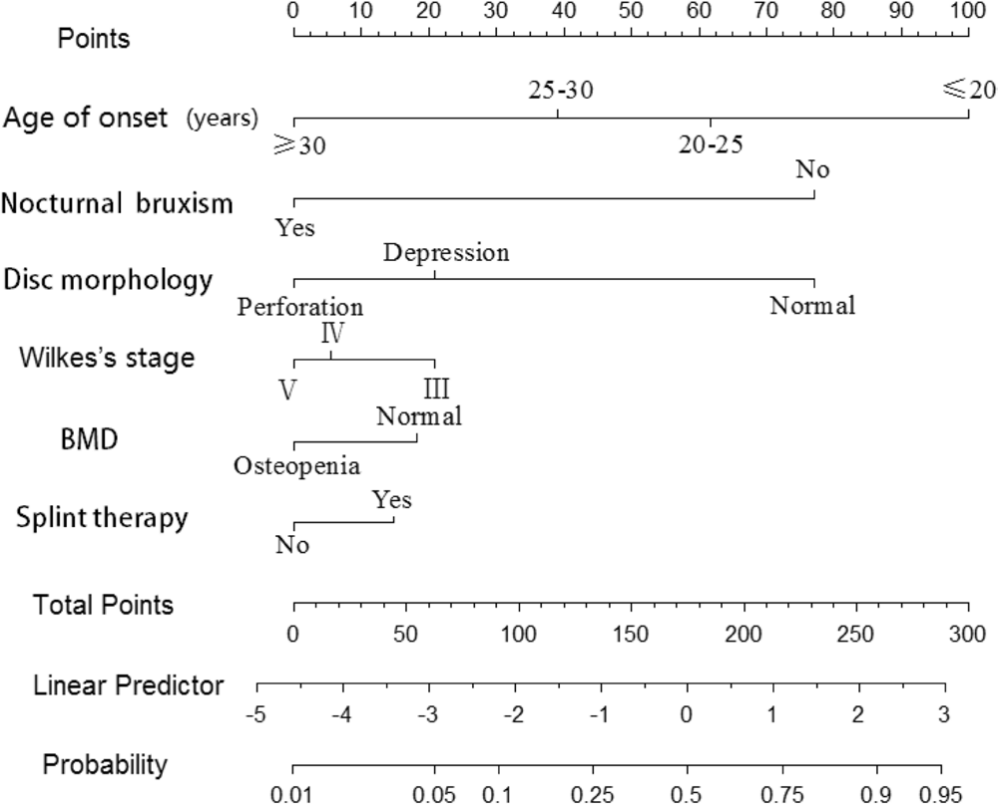
Prognostic nomogram for postoperative bone remodeling in patients with ADDWoR. (The individual patient’s value is located on each variable axis, and a line is drawn upward to determine the number of points received for each variable value. The sum of these numbers is located on the Total Points axis, and a line is drawn downward to the probability axes to determine the post-operative bone remodeling). Abbreviations: BMD, bone mineral density.

A nomogram that incorporated the significant prognostic factors was established in Fig. 2. In the nomogram, points were assigned to all variable factors, and a sum of points within the factors were depicted on the total point scale. A straight line could then go down to determine the estimated probability of post-operative bone remodeling (linear predictor, probability) at each time point. In this study, the higher score of total points represented for a more probability of bone regeneration^11^. The result of the nomogram illustrated that the age of onset shared the largest contribution to postoperative condyle modification, followed by the nocturnal bruxism experience. The factors of Wilkes’ classification, disc morphology, condyle situation and the post-operative splint therapy showed a moderate to low impact on the result. In the primary setting, the ROC curve of discrimination for the established nomogram was good, with the AUC of 0.84 (95% CI, 0.80 to 0.89) after bias correction with 2,000 cycles of bootstrap resampling. The calibration plots also presented with a good agreement on the nomogram prediction and actual observation in the probability of post-operative bone remodeling (Appendix Fig. 1).

## Methods

### Patients and Study Design

A retrospective cohort study consisting of 1110 patients with ADDWoR treated by arthroscopy surgery at the Shanghai Ninth People’s Hospital, Shanghai Jiao Tong University (Shanghai, China) was conducted from January, 2007 to January, 2017.

The inclusion criteria were as follows: (1) diagnosed of disc displacement without reduction; (2) treated by arthroscopy therapy; (3) with complete preoperative and postoperative MRI assessment. Exclusion criteria included patients with uncertain diagnose, with poor quality of MRI imaging, without complete data sheet or loss to follow-up.

The study was approved by the Ethics Committee of Shanghai Jiao Tong University School of Medicine and all participants (primary and validation cohort) provided informed participation consent in accordance with the relevant regulations and guidelines.

### Diagnosis and Treatment

A routine investigation including personal information (age, gender) and detailed history (course of disease, nocturnal bruxism) were taken from all patients. Then examinations involving clicking, pain-related disorders, maximum interincisal opening (MIO), and bone mineral density (BMD) were taken into consideration^13^. In our study, the condyle condition (BMD) was composed of two indexes. According to the International Society of Clinical Densitometry (ISCD), the value of the lumbar spine (anterior-posterior at L2–L4) and proximal femur (femoral neck, trochanter, and Ward’s triangle) was measured using dual-energy X-ray absorptiometry (DEXA, DMS Lessos, France) in which a Z-score ≤ −2 was termed as low^14,15^. Besides, a condyle osteopenia was also measured using MRI examination in which a hypointense signal instead of a normal hyperintense signal was detected on T1 images of bone marrow area. The ‘osteopenia’ changes were required to present on at least two consecutive slices^14,16^.

The preoperative diagnosis of stage of ID was evaluated in relation to the Wilkes-Bronstein classification based upon clinical and MRI examination^17^. According to the classification, ADDWoR was reported in Stage III (serious jaw functional obstacles, chronic pain, and mild to moderate disc deformity, Stage IV (serious jaw functional obstacles, chronic pain, co-occurring with severe disc posterior band hypertrophy, and abnormal bone structure), and Stage V (serious jaw functional obstacles, chronic pain, obvious disc deformity, disc perforation, and degenerative bone changes)^8^.

Meanwhile, arthroscopy surgery was carried out for patient who was diagnosed of ADDWoR, complained of pain and/or dysfunction and failed to conservative therapies^8,18^. All treatments were performed according to the guidelines for TMD treatment and carried out by the same surgeon (with more than 40 years of experience in TMJ treatment)^19^. The surgical procedure was commonly involved three main parts including anterior release, disc reduction, and disc suture^10^. For all patients included, the technique began by local anesthesia (2% lidocaine 2 to 3 ml) injected into the superior joint space to avoid pain and to decrease bleeding. Firstly, a puncture aimed at a systematic diagnostic arthroscopy was injected on the top of the fossa. For anterior release, the second puncture located at the anterior surface of the eminence. Under this puncture, a coblation probe was inserted in and an incision from medial to lateral was carried out 2–3 mm anterior to the anterior band of the disc. To avoid the injury of both blood vessels and nerves, the depth of the anterior release was recommended to limited under 2 mm. After that, an obturator was applied for disc reduction. For disc suture, body projection was commonly 10 mm ahead of the first puncture. Under the direct visualization of arthroscopy, a 12-gauge suturing needle was injected into the border of the bilaminar zone and the posterior band. The needle was pushed in and out of the retrodiscal tissue directed from medial to laterial. Meanwhile, another puncture was performed through the point at the anterior wall of the external auditorycanal. Through these two punctures, customized suture grippers was inserted to hold surgical sutures. Finally, sutures were tied with the knots underneath the cartilage of the external auditory canal and the skin incision was closed. Then a 6 to 12 month post-operation functional splint treatment was conducted if an open bite over 2 mm was detected in patients. All patients were scheduled a complete physical examination at each of the follow-up visits (1, 3, 6, 12, 18, 24 month). A MRI was also performed once every 6 months in the following 2 years^20^.

**Figure 3:**
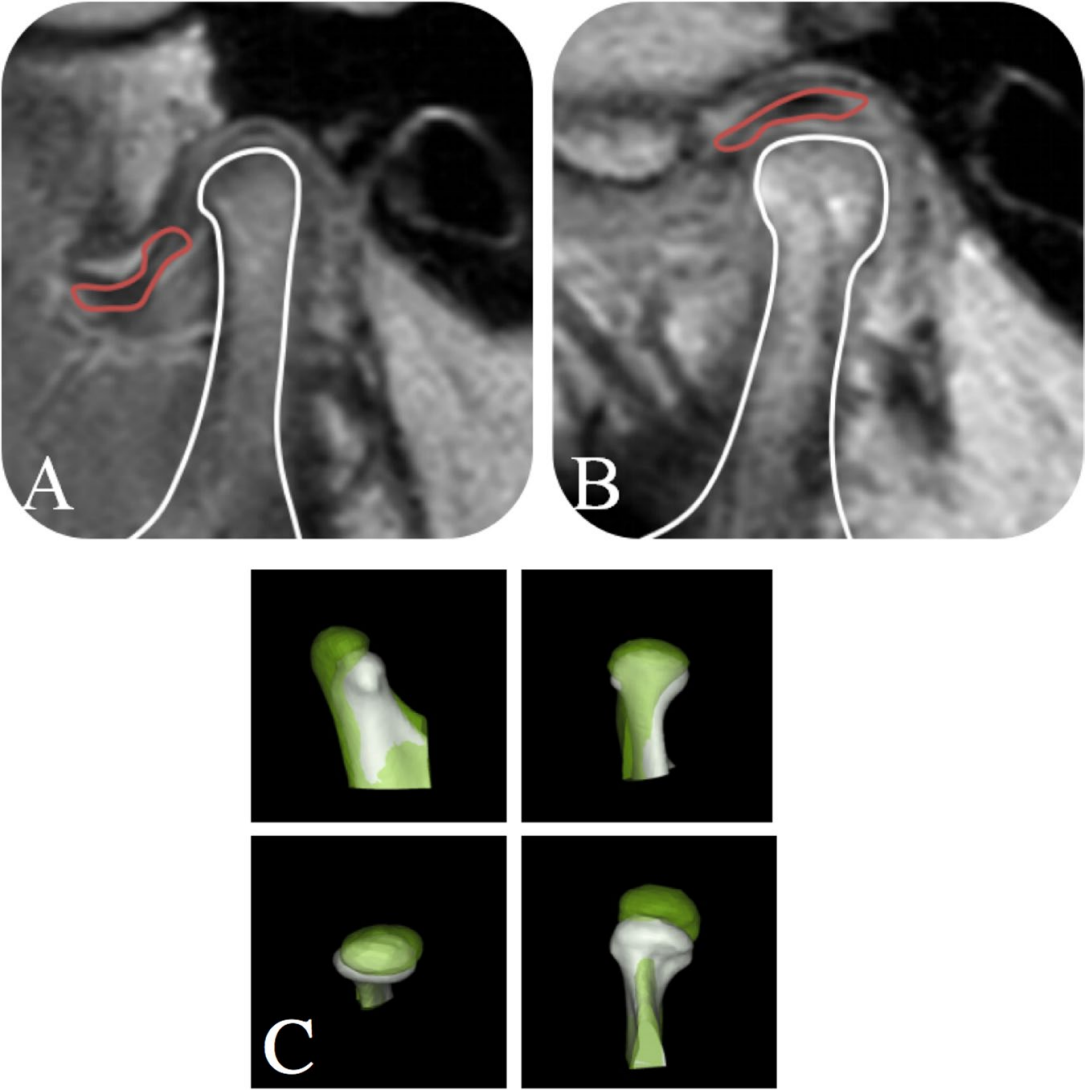
MRI of ADDWoR before treatment (**A**); MRI of ADDWoR 12 months after arthroscopy surgery (**B**); 3D volume-rendering images showed achievement of vertical and horizontal bone augmentation (**C**).

Bone modification was evaluated upon pre- and post-operative MRI, in which either a smooth and continuous cortical bone in the condylar sagittal appearance and/or new bone tissue characterized by a mass hyperintense signal intensity on laterial of condyle (T1-and T2-weighted spin echo) was observed when compared with the baseline (Fig. 3A,B)^16^. In the study, all scans were executed at the Shanghai Ninth People’s Hospital, Shanghai Jiao Tong University (Shanghai, China) by the same technician. Two independent readers reviewed each MRI of patient blindly in parallel. Any discrepancy was resolved through a discussion with a third investigator (C.Y, with more than 30 years of experience in oral surgery). In some cases, a 3D volume-rendering image based on CT scan was used to calculated the changes of condyle remodeling after the disc reposition treatment. In this study, a method of image fusion was carried out by using a multipoint registration^21^. According to landmark points (the genial tubercle, the mental foramen, the inferior loop of alveolar nerve, and the sigmoid notch), post-operative images in 12 months were moved to coordinate to the pretreatment 3D model voxel-by-voxel^22,23^ (Fig. 3C).

### Statistical Analysis

Categorical variables grouped upon clinical and radiological findings are set in proportions. The result was evaluated by the X^2^ test or the Fisher’s exact test. Continuous variables were also compared under the t test for variables distribution. Logistic regression analysis was carried out for the multivariate analysis. Variables were formulated on a backward step-down model reduction, with the elimination criterion of P > 0.05^24^. The nomogram was then programmed based on the result of multivariate analysis. Performance was measured by operating characteristics curve (ROC) associated with AUC (area under the ROC curve) for discrimination^25^. Calibration was performed by comparing the predicted bone remodeling with the observed subject after bias correction in accordance with the calibration curve. According to the established nomogram, bootstraps with 2,000 resample were used in internal and external validation for average bias. The statistical analyses were conducted using R software with rms-package (http://www.r-project.org).

## Discussion

Disc displacement in TMJ is a common disruption condition causing progressive joint dysfunction. Longitudinal clinical observations of orthodontics, orthognathics, and articulationes mandibularis suggested that disc displacement, in general, is associated with mandibular asymmetry^1,2^. In Hall *et al*. study, it has been deduced that disc displacement may give rise to condylar degeneration thus causing the bone resorption and the decrease of condylar height among patients^5^. For patients with unilateral ADDWoR, the growth of affected condyle was restricted, while the other side developing properly, and which may finally give rise to mandibular deviation. In the patients involving bilateral sides, accordingly, the development of mandible was obviously restrained. Instead of the normally forward and counterclockwise growth, a backward and clockwise trend was detected, thus causing mandibular retrusion. In addition, Cai *et al*. discovered that compared with short-term disc displacement, the length of disc was shorter with the distance of displacement getting farther and the height of condyle decreased in long-term cases^26^. As for patients of disc replacement, the operation of disc reposition remains estimated. According to Yang *et al*., it is unnecessary to have disc reposition in stable cases with no clinical symptoms, which includes patient of relatively completed growth, the integrity condylar cortical on MRI, no articular degeneration of joint, disc-like-change in bilaminar zone, and no clinical and imaging alternation of joint and maxillofacial during the interval of more than 6 months^19^. In Wilkes’ stage evaluation, similarly, invasive intervention treatment was recommended especially in those who have significant clinical symptoms such as pain, dysfunction, induced mandibular deviation, mandibular retrusion or failed to conservative therapies^8^.

During longitudinal follow-up, studies found that disc reposition can prevent the further resorption and degeneration of the condyle and produce adaptive amelioration as new bone formation. Especially in young patients with positive bone marrow quality, it is common to observe condylar regeneration^27^. This postoperative reconstruction could bring the increase of the height of the affected condyle for patients with unilateral ADDWoR, and therefore improve the facial and occlusal deviation. In the patients with bilateral defect, mandibular retraction (type II) will be corrected (type I) or improved with the advancement of mandibular.

As for the postoperative evaluation, potential factors involving age, gender, genetic background, nutritional status, repetitive oral habits, and treatment strategy have all been cited as trigger or aggravator^11^. However, none of the parameters have been specifically developed for the postoperative prognostic prediction. Our study was therefore to evaluate the condylar reconstruction after disc reposition treatment and its related factors.

A prognostic nomogram model for patients with ADDWoR treated by surgical approaches was constructed using post-operation bone remodeling as established curative modality. The AUC value of 0.84 calculated for the nomogram model indicated a high prognostic prediction accuracy.

In this cohort study, a multivariate logistic regression analysis was performed among potential factors including age of onset, course of disease, BMI, nocturnal bruxism, pain, disc morphology and BMD, and post-operative splint therapy. Our study detected a predominace in the parameter of age among other factors. Lower post-operative bone regeneration rate was found in aging population, with less than 50 points for age over 25 years old. Evidence suggested that the decrease in intrinsic properties of mesenchymal stem cells (MSCs) which compromise to bone remodeling, especially to bone loss are age-related^28^. According to Goncalves *et al*., a continuous and compact cortical bony layer was established at the age of twenties, which indicated a full development of the mandibular condyle^29^. In relevant, a complete cortical bony formation was also seen at the average age of 22 years for male and 21 for female. Meanwhile, osteogenic/adipogenic differentiation, and osteoclastogenesis activity also changed greatly during the aging process. Accordingly, the age-dependent growth of homocysteine in blood levels may lead to the potential decline in BMD among elder patients. Associated with BMD degeneration, the negative correlation also affects the viscosity bone pathology, which is responsible for impaired bone regeneration^30^. And therefore, superior bone remodeling was detected in younger group underwent earily treatment. In this study, condyle condition (BMD) was ranked as a factor in post-operative bone remodeling.

According to Liu *et al*., articular disc perforation occurred more frequently in anterior disc displacement without reduction, especially in the bilaminal zone. In our study, among 1110 joints, nearly 15.23% reported of disc perforation. Moreover, TMJ disc perforation has also been confederated in patients with heterotopic ossifications (HO), and rheumatic/inflammatory disease. Based on histological analysis, pathological features such as the earily formation of chondrocyte clusters, the thickening of cartilages, and the multifocal loss of cells were detected in condyle surface, which may then blemish on the subchondral bone, causing a diffuse loss of cells and proteoglycan content^31^. Besides, inflammatory mediators may come to assemble in perforation site causing synovial inflammation and resulting in pain and new bone erosion. Our study demonstrated that a high-quality disc trended to result in large improvements in the postoperative bone remodeling, with a mean improvement of 76 points at the two-year follow-ups. Thus, it was encouraged a well maintenance of disc condition in early time.

Furthermore, mechanical forces such as chewing and bruxism are other major factors in postoperative bone absorption. Li *et al*. analyzed the effect of pressure change on condyle and chondrocytes based on the model of rabbits^32^. The results showed that under the pressure of more than 200KPa, the proliferative ability of condylar chondrocytes decreases, with the necrosis and apoptosis increase significantly, which indicate that increasing pressure disturbs the proliferation of chondrocytes in condylar cartilage, and lead to the cartilage degeneration^33^. Occlusion splints, however, are effective in releasing TMJ involuntary overloading and to reduce the muscle hyperactivity. Moreover, a subsequent occlusion reconstruction achieved by postoperative splints can help to maintain an optimal environment within the TMJ, thus leading to bone osteogenesis and reducing relapse rate^34^. In our study, almost 610 patients have the habit of nocturnal bruxism and 345 patients took functional splint treatment after arthroscope surgery with 48.51% detected of bone remodeling. In recent research, functional splints have also been recommended with additional effect on the improvement of psychological aspects in TMDs^35^. Meanwhile, other factors involving long-term progress with pain and clicking are negative predictors in postoperative bone modification.

This is the first nomogram for predicting post-operative bone remodeling of patients with ADDWoR. The analysis was based on a large database with long-term follow-up. Multivariate analyses including age of onset, nocturnal bruxism, the Wilkes’ stage classification, disc morphology, BMD, and post-operative splint therapy were all taken into consideration. In order to enhance the accuracy and reliability of the findings, data extraction, and statistical analysis were all performed by two investigators independently. ROC curve (AUC-index) and calibration curve were calculated in evaluating predictive accuracy and discriminative ability.

Still, limitations in current nomogram must be considered. The study was established based on data obtained from a single institution and some potential prognostic parameters such as geographic and ethnic difference were failed to be incorporated. Therefore, further efforts on prospective data collection of multicenter are encouraged to improve this model. Besides the assessment of post-operative bone remodeling was primarily based on the MRI imaging. Although the diagnostic accuracy of MRI in detecting the TMJ is reported over 86%, there still existed a limitation to a certain extent. Thus, an enhanced CT evaluation was recommended in later research.

In conclusion, a novel nomogram for predicting post-operative bone remodeling of patient with ADDWoR was validated, which provided an objective and accurate individualized post-operative prediction for both physicians and patients.

## Electronic supplementary material

Appendix Figure 1
