# Supplementary material for: A Prognostic Nomogram for Postoperative Bone Remodeling in Patients with ADDWoR
Source: Sci Rep. 2018 Mar 12;8:4361. doi: 10.1038/s41598-018-22471-x (PMC5847608; doi:10.1038/s41598-018-22471-x)

**A Prognostic Nomogram for Postoperative Bone Remodeling in Patients with ADDWoR**

Xiaohan Liu^1,2^*, Pei Shen^2^*, Xiangyu Wang^2^, Shanyong Zhang^2^, Jiawei Zheng^1,3,4#^, Chi Yang^2#^

1. College of Stomatology, Shanghai Jiao Tong University School of Medicine, Shanghai, China.

2. Department of Oral Surgery, Ninth People’s Hospital, Shanghai Jiao Tong University School of Medicine.

3. Department of Oral-Maxillofacial Head and Neck Surgery, Ninth People's Hospital, Shanghai Jiao Tong University School of Medicine.

4. Shanghai Key Laboratory of Stomatology & Shanghai Research Institute of Stomatology; National Clinical Research Center of Stomatology.

* These authors are co-first authors.

# Correspondence and requests for materials should be addressed to ：

C.Y. Department of Oral Surgery, Ninth People’s Hospital, Shanghai Jiao Tong University School of Medicine, 639 Zhizaoju Road, Shanghai, China, 200011 Email: [yang_chi63@163.com](mailto:yang_chi63@163.com) or JW.Z. Department of Oral and Maxillofacial Surgery, Ninth People’s Hospital, Shanghai Jiao Tong University School of Medicine, 639 Zhizaoju Road, Shanghai, China, 200011 Email: [davidzhengjw@sjtu.edu.cn](mailto:davidzhengjw@sjtu.edu.cn).

Appendix Figure 1. The ROC curve (A) and calibration curve (B) for predicting postoperative bone remodeling in patients with ADDWoR.


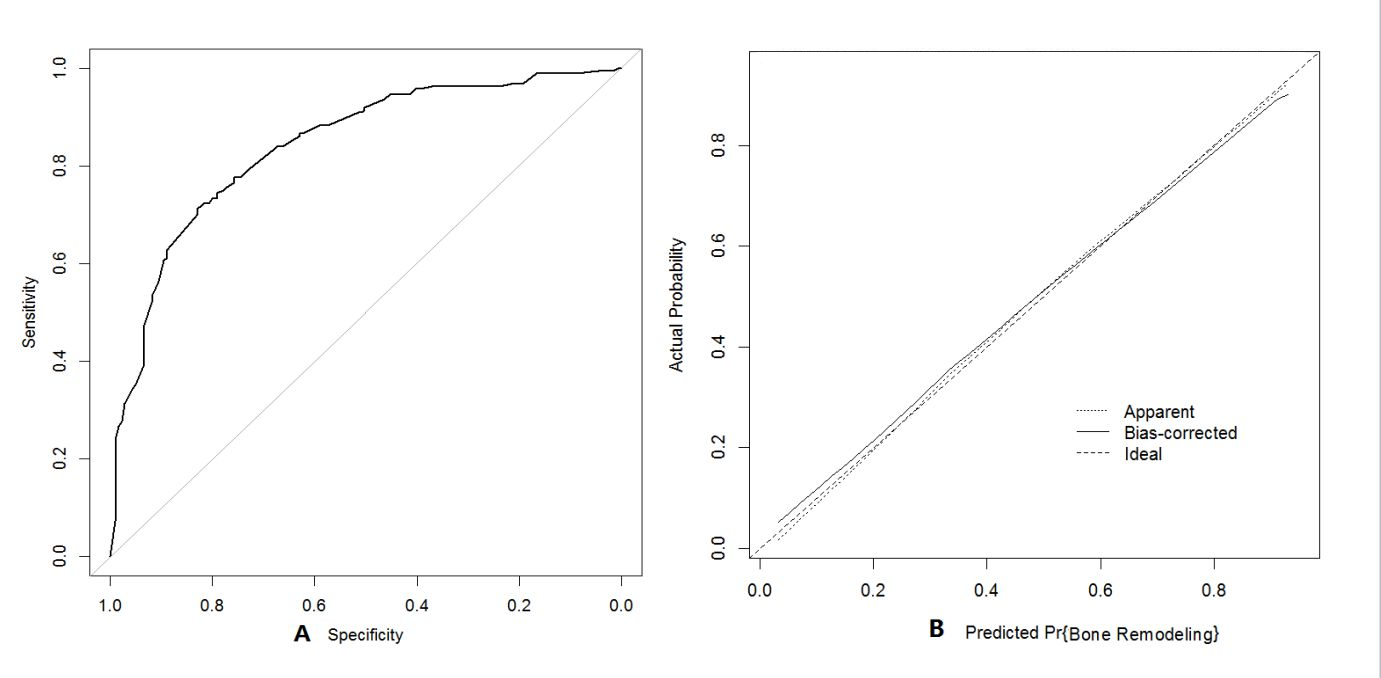

Supplement: Supplementary file 1 — Appendix Figure 1 [file 41598_2018_22471_MOESM1_ESM.docx]
